# Primary Hepatic Leiomyoma in a Healthy Middle-Aged Woman: Literature Review and Case Report

**DOI:** 10.3389/fsurg.2021.691674

**Published:** 2021-06-14

**Authors:** Mihajlo Djokic, Benjamin Hadzialjevic, Bostjan Luzar, Blaz Trotovsek

**Affiliations:** ^1^Department of Abdominal Surgery, University Medical Centre Ljubljana, Ljubljana, Slovenia; ^2^Department of Surgery, Faculty of Medicine, University of Ljubljana, Ljubljana, Slovenia; ^3^Institute of Pathology, Faculty of Medicine, University of Ljubljana, Ljubljana, Slovenia

**Keywords:** leiomyoma, liver, neoplasm, primary hepatic leiomyoma, surgery, immunohistochemistry

## Abstract

**Introduction:** Primary hepatic leiomyoma (PHL) is a rare benign hepatic tumor with unclear pathogenesis. It more commonly occurs in immunosuppressed patients, while only 24 cases have been described among immunocompetent individuals. To date, only one successful preoperative diagnosis of PHL has been achieved.

**Case Presentation:** Here we report a case of PHL in a middle-aged woman with no history of immunosuppression. Preoperative diagnosis of PHL was established using ultrasound-guided fine needle trucut biopsy (FNTB). Nevertheless, due to the growing nature of tumor and patient's symptoms, we proceeded with surgical resection, which confirmed the diagnosis of PHL. At 6-month follow up, the patient is in good condition with no evidence of tumor recurrence.

**Conclusions:** PHL is an uncommon tumor that should be considered in the differential diagnosis of rare liver tumors. Image guided FNTB appears to be effective in achieving preoperative diagnosis of PHL. Surgical resection, however, remains both diagnostic and curative in the management of PHL.

## Introduction

Leiomyoma is a benign mesenchymal tumor with smooth muscle differentiation that commonly occurs in genitourinary system, particularly uterus, and gastrointestinal tract (1). Primary hepatic leiomyoma (PHL), however, is very rare. It was first described in 1926 and since then, most of the cases have been reported in immunocompromised patients (2, 3). To date, only 24 cases have been described among immunocompetent individuals.

Here we report a case of PHL in an otherwise healthy, middle-aged female with no history of immunosuppression.

## Case Presentation

**Figure 1:**
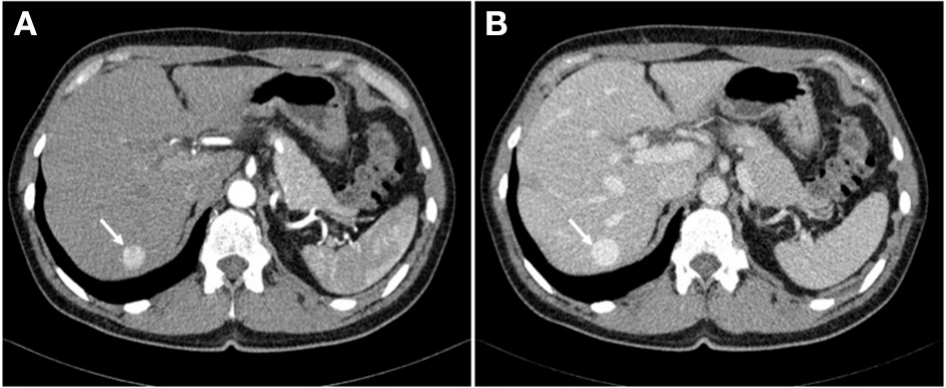
Abdominal CT scan demonstrating a 15 mm hypervascular lesion in segment VII of the liver in arterial phase **(A)** and venous phase **(B)**.

A 56-year-old Caucasian female was referred to Clinical Department of Abdominal Surgery, University Medical Center Ljubljana for further investigation and treatment of a growing intrahepatic mass. Initial abdominal ultrasound (US), which had been carried out 6 years previously for evaluation of nephrolithiasis, revealed a hypoechoic lesion in a segment VII of the liver. Further contrast-enhanced computed tomography (CT) scan confirmed a 15 mm hypervascular lesion in segment VII (Figure 1). Periodic follow-ups during the subsequent years revealed a progressive increase in the size of the lesion, which by the time of her referral in had grown for more than 30 mm, and reached the size of 49 mm.

In her past medical history, she underwent laparoscopic cholecystectomy due to acute calculous cholecystitis. She had no history of chronic illnesses and she took no medications. She reported intermittent pain in the right upper quadrant, which had started few weeks before the admission. Physical examination was unremarkable. Laboratory results including tumor markers alpha-fetoprotein (AFP), carcinoembryonic antigen (CEA), and cancer antigen 19-9 (CA 19-9) were all within normal ranges.

**Figure 2:**
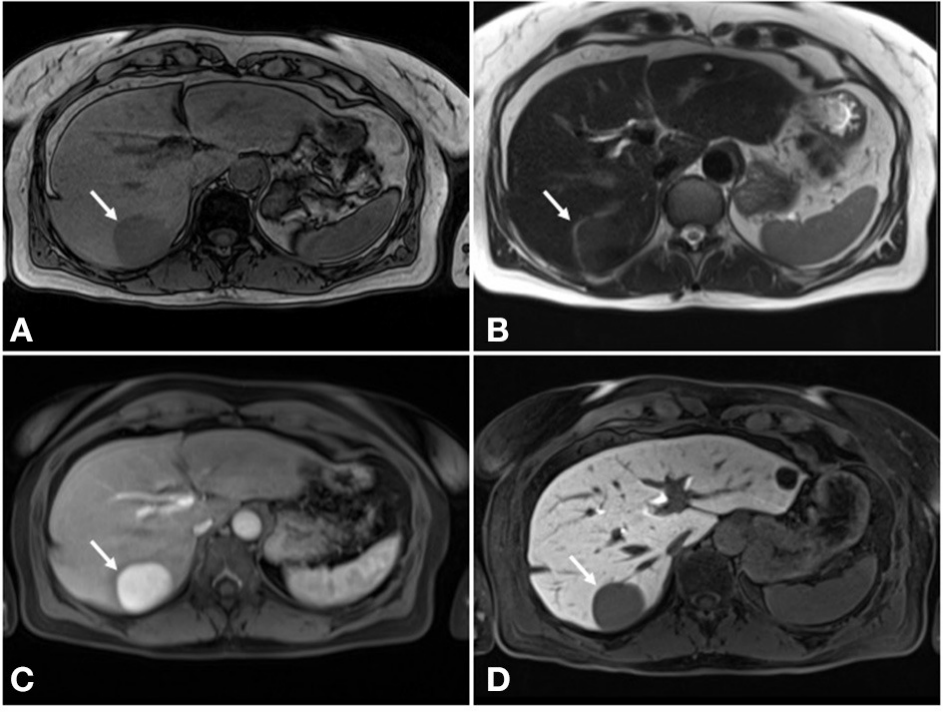
MRI of the liver. **(A)** Hypointense signal on T1-weighted sequences. **(B)** Hyperintense signal on T2-weighted sequences. **(C)** Complete homogenous enhancement in post-contrast arterial phase. **(D)** Hypointense signal in hepatobiliary delayed phase.

Contrast-enhanced US showed a hypoechoic lesion with hyperenhancement in arterial phase that progressed from the periphery to the center and subsequent washout in the portal-venous phase. Further magnetic resonance imaging (MRI) demonstrated the same lesion, which was better defined as a 45 × 49 mm round lesion in segment VII with a hypointense signal on T1-weighted sequences and hyperintense signal on T2-weighted sequences with complete homogenous enhancement in post-contrast arterial phase and hypointense signal in hepatobiliary delayed phase (Figure 2). These findings suggested portal vein aneurysm, arteriovenous aneurysm, or hepatocellular adenoma in the differential diagnosis. Additionally, MRI showed three hepatic cystic lesions, which measured between 9 and 20 mm.

Since imaging studies were inconclusive, US-guided 18G fine needle trucut biopsy (FNTB) was performed. Biopsy specimen, measuring about 50 × 1 mm, revealed a benign mesenchymal tumor composed of bland spindle cells forming short fascicles. The nuclei of tumor cells were elongated and often cigar shaped lacking mitotic activity, while the cytoplasm was moderately abundant and eosinophilic. By immunohistochemistry, tumor cells were positive for smooth muscle actin, desmin, H-caldesmon and calponin, and were negative for CD117, DOG1, STAT6, MUC4, and wide spectrum cytokeratins. Based on the morphological features and supplemented by additional immunohistochemistry, a diagnosis of a benign smooth muscle tumor was suggested, consistent with leiomyoma.

Patient's records were presented at a hepatobiliary multidisciplinary team meeting. As tumor had grown progressively to a size of almost 50 mm and the fact that it became symptomatic, surgical removal was recommended. Thus, laparotomy was carried out in an usual manner. Surgical exploration and intraoperative US revealed a 49 × 41 mm solid mass in segment VII of the liver in close proximity to the right hepatic vein. Resection of segment VII (i.e., segmentectomy) was performed using cavitron ultrasonic surgical aspirator (CUSA) (Integra Life Sciences, NJ, USA). Furthermore, two simple hepatic cysts in segment II were also removed. Finally, a thorough abdominal and pelvic exploration with gastric and small bowel palpation was performed, all of which were unremarkable. The postoperative period was uneventful and the patient was discharged on the fifth day after surgery.

**Figure 3:**
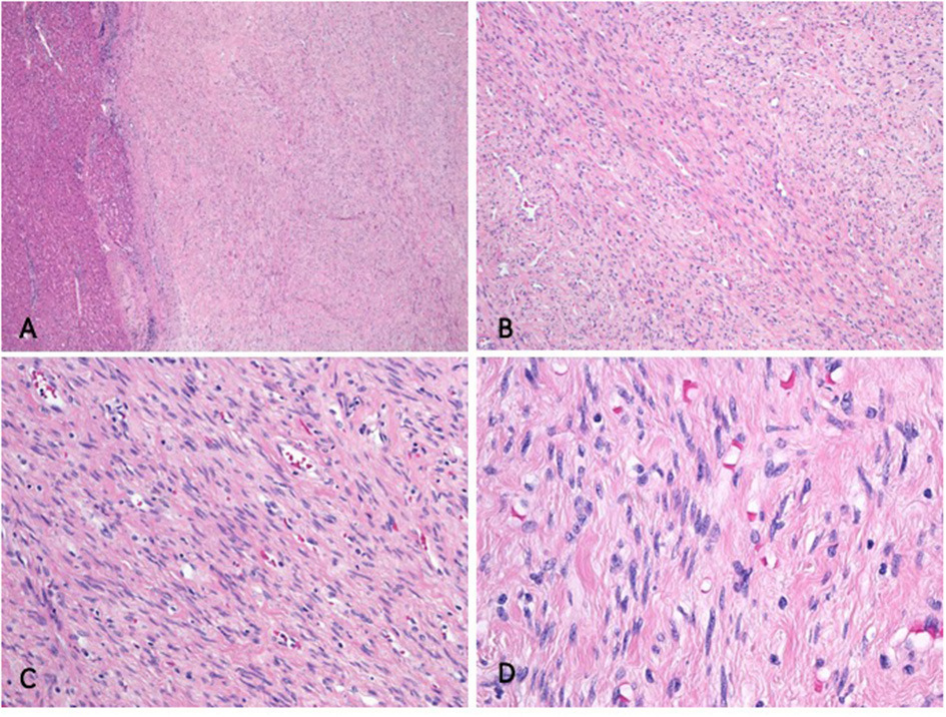
Histologic analysis. **(A)** The well-demarcated and unencapsulated tumor is seen in the liver. **(B)** The tumor is composed of fascicles featuring cells with bland, elongated cigar shaped nuclei and eosinophilic cytoplasm. **(C)** Higher magnification depicting bland tumor cells with cigar shaped nuclei and indistinct eosinophilic cytoplasm. Note the absence of mitotic activity. **(D)** Palisading of the nuclei was present at many tumor foci.

**Figure 4:**
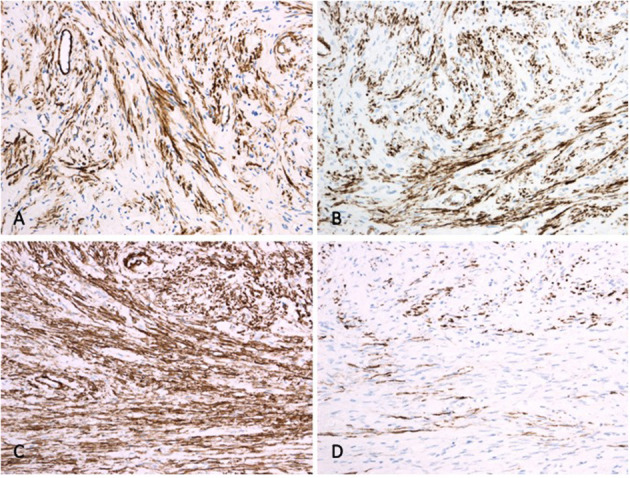
Immunohistochemistry. While tumor cells are diffusely positive for calponin **(A)**, h-Caldesmon **(B)** and smooth muscle actin **(C)**, only about 60% of the tumor cells display desmin positivity **(D)**.

Histologic analysis revealed a well-demarcated and unencapsulated tumor within the liver (Figure 3A), composed of fascicles of bland tumor cells with elongated cigar shaped nuclei and eosinophilic cytoplasm with indistinct borders (Figures 3B,C). Mitotic activity was very low (<1 mitosis per 10 high power fields), atypical mitoses were absent. Focal palisading of the tumor cell nuclei was present at many areas (Figure 3D). The surrounding liver parenchyma was unremarkable. By immunohistochemistry, the tumor cells were diffusely positive for h-Caldesmon, calponin, and smooth muscle actin, while only about 60% of tumor cells displayed strong positivity for desmin (Figure 4). The tumor cells were negative for S100, CD34, MUC4, STAT6, C-KIT, DOG1, and CK-MNF116. *In situ* hybridization was negative for Epstein-Barr virus (EBV). In summary, morphological and immunohistochemical features were consistent with hepatic leiomyoma. An extensive clinical work-up failed to detect the presence of smooth muscle neoplasm outside the liver, including gastrointestinal and genitourinary tract. Therefore, a final diagnosis of a PHL was established.

At 6-month follow up, the patient is in an excellent clinical condition, with no evidence of recurrence. She continues to adhere to surveillance at every 6 months.

## Discussion

PHL is an uncommon, benign hepatic tumor that affects both children and adults with slight female predominance (2). To diagnose PHL, Hawkins et al. (4) proposed two criteria. Besides the tumor being composed of smooth muscle cells, distant leiomyomas elsewhere in the body must be excluded. Since it was first described in 1926 by Demel et al. (5), less than 50 cases of PHL have been reported in medical literature with only 24 cases among immunocompetent patients (6).

The pathogenesis of PHL is not well-understood. Although, it was proposed that PHL can originate from smooth muscle cells of hepatic vessels or bile ducts, this has not been proven in later studies (7, 8). In general, smooth muscle tumors occur with a greater frequency among human immunodeficiency virus (HIV) positive patients and post-transplant patients on immunosuppressive therapy, mostly in combination with EBV infection (9, 10). Thus, these factors have been implicated in the pathogenesis of PHL since EBV has been detected in PHL of immunocompromised patients (11, 12). However, as in our case, PHL can also arise in healthy patients. Therefore, immunosuppression and EBV infection do not completely explain pathogenesis of this disease, which remains complex and multifactorial (1).

Patients with hepatic leiomyoma are often asymptomatic, or they have non-specific symptoms that are similar to those of other benign liver tumors. The most common complaint among symptomatic patients is right upper quadrant pain followed by epigastric pain and diffuse abdominal pain (3). In two cases hepatic leiomyoma presented with dyspepsia and liver dysfunction (2).

The widespread use of imaging techniques has led to an increased detection of incidental liver tumors. Since differential diagnosis of liver incidentalomas is extremely broad, rare liver tumors remain a diagnostic challenge for most clinicians (13). On US, PHL have appeared as hypoechoic solid lesion with varying degrees of heterogeneity (14, 15). On contrast-enhanced US, authors demonstrated a marked peripheral contrast enhancement (16). Reported contrast-enhanced CT features include hypodense lesions with marked, mainly peripheral enhancement in the arterial through portal and delayed phases (15, 17). On MRI scans, lesions usually appear hypointense on T1-weighted images and hyperintense on T2-weighted images with marked gadolinium enhancement in both early and equilibrium phases (15, 18). However, T2-weighted hypointense lesions have also been reported, which were associated with dense fusocellular nature of the tumor (14). Our findings were consistent with previous reported studies. In addition, on contrast-enhanced US, we demonstrated a hyperenhancement in arterial phase that progressed from the periphery to the center and subsequent washout in the portal-venous phase.

Imaging modalities do not exhibit tissue-specific diagnosis, therefore final diagnosis is ultimately achieved after histologic examination of the surgical specimen. Nevertheless, several attempts at achieving preoperative diagnosis *via* interventional/percutaneous methods have been described. To date, only one successful preoperative diagnosis of PHL has been reported by Sousa et al. (14). At first, they were unsuccessful with US-guided fine needle aspiration (FNA) due to insufficient material, which was consistent with previous and later studies (6, 19). Subsequently, however, they managed to achieve preoperative diagnosis of PHL with a 18G FNTB of the liver lesion (14). Similarly to their case, we also achieved preoperative diagnosis of PHL using an US-guided FNTB. To the best of our knowledge, this is the second report of successfully established preoperative diagnosis of PHL.

Surgical resection has been demonstrated to be both diagnostic and curative in the management of hepatic leiomyoma. Right hepatectomy, left hepatectomy, or segmentectomy, either *via* an open or laparoscopic approach, have all been shown as safe and feasible in the treatment of hepatic leiomyoma (14, 17). The vast majority of patients have been managed using an open liver resection with only few reports of laparoscopic approaches for hepatic leiomyomas, all of which were located in peripheral liver segments (3, 18, 20). Generally, surgical site infections and incisional hernias occur with higher frequencies in immunocompromised patients, in which PHL is also more common. Therefore, minimally invasive liver resection (either laparoscopic or robotic) could be particularly beneficial in these patients as it has been associated with lower rates of postoperative wound complications (6, 18). Nonetheless, since tumor was located in posterolateral segment of the liver, we chose to proceed with open liver resection. PHL appears to have an excellent prognosis as no recurrences of the disease have been reported so far. Postoperative follow-ups ranged from 4 to 108 months (2). Even though there is no consensus regarding the frequency, duration and mode of follow-up for PHL, each case should be presented to a hepatobiliary multidisciplinary team for the individual decision of postoperative surveillance for these patients.

Histological examination can differentiate between benign and malignant smooth muscle tumors (8). It also represents one of two criteria for the diagnosis of PHL proposed by Hawkins et al. (4). In general, findings such as high mitotic index, prominent cellular atypia, areas of coagulative tumor cell necrosis, dense cellularity, nuclear pleomorphism, and large size of tumor are indicative for malignant smooth muscle tumor (18, 21). Hepatic leiomyoma is typically characterized by bundles of smooth muscle cells and eosinophilic cytoplasm (1, 20). Focal areas of necrosis and scarce or rare mitotic figures have also been reported (8, 11, 18). A recent study on gastrointestinal smooth muscle tumors, including esophageal, gastric, small bowel, and colorectal tumors suggested a novel approach in classifying these tumors according to progression risk, akin to gastrointestinal stromal tumors (GIST) into low risk, intermediate risk and high risk groups based on the location of the tumor, size, and frequency of mitotic activity (22). Nevertheless, such an approach has not yet been validated for primary smooth muscle neoplasms of the liver.

The main histological differential diagnosis of PHL includes other primary spindle cell tumors in the liver, either metastatic or primary. Distinction can prove extremely difficult especially in small biopsy samples. Immunohistochemistry is a useful tool to differentiate between hepatic leiomyoma and potential mimickers, and can be supplemented by additional molecular genetic testing whenever necessary. Only GIST, solitary fibrous tumor and low grade fibromyxoid sarcoma will be discussed herein. GIST is characterized by C-KIT and Dog-1 positivity and can be further confirmed by molecular genetic testing to detect *KIT* or *PDGFRA* gain-of-function mutations or aberrations in the succinate dehydrogenase gene (23, 24). A solitary fibrous tumor will usually reveal STAT6 nuclear positivity by immunohistochemistry and a characteristic recurrent *NAB2-STAT6* fusion by molecular genetic testing (25). Primary hepatic low grade fibromyxoid sarcoma is an exceedingly rare tumor (26). While positive MUC4 immunohistochemistry in this tumor is a very sensitive marker, molecular genetic testing to confirm rearrangement of the *FUS* gene is diagnostic of the entity (27).

## Conclusion

PHL is an extremely rare, benign hepatic tumor that should be considered both in immunocompromised and immunocompetent patients. As imaging modalities do not exhibit tissue-specific diagnosis, histologic examination of specimen is essential for diagnosis. In the absence of distant leiomyomas, image-guided FNTB appears to be effective in achieving preoperative diagnosis of PHL. Surgical resection, however, remains both diagnostic and curative in the management of PHL.

## Data Availability Statement

The original contributions presented in the study are included in the article/supplementary material, further inquiries can be directed to the corresponding author/s.

## Ethics Statement

Ethical review and approval was not required for the study on human participants in accordance with the local legislation and institutional requirements. The patients/participants provided their written informed consent to participate in this study. Written informed consent was obtained from the individual(s) for the publication of any potentially identifiable images or data included in this article.

## Author Contributions

BT has been involved in the management of the patient. BL contributed to the pathological diagnosis. BH wrote the first manuscript. MD, BL, and BT assisted in the preparation of the manuscript. All authors have read and approved the final manuscript.

## Conflict of Interest

The authors declare that the research was conducted in the absence of any commercial or financial relationships that could be construed as a potential conflict of interest.
